# Treatment outcomes for multidrug-resistant tuberculosis under DOTS-Plus: a systematic review and meta-analysis of published studies

**DOI:** 10.1186/s40249-016-0214-x

**Published:** 2017-01-17

**Authors:** Kelemu Tilahun Kibret, Yonatan Moges, Peter Memiah, Sibhatu Biadgilign

**Affiliations:** 1 Department of Public Health, College of Medical and Health Sciences, Wollega University, P. O. Box 395, Nekemte, Ethiopia; 2 Department of Public Health, College of Medical and Health Sciences, Haramaya University, P. O. Box 135, Harar, Ethiopia; 3 College of Health, Department of Public Health, University of West Florida, Florida, USA; 4 Public Health Research Consultant, P. O. Box 24414, Addis Ababa, Ethiopia

**Keywords:** Tuberculosis, Multidrug resistance, DOTS-Plus, Multidrug-resistant tuberculosis, Treatment outcomes

## Abstract

**Background:**

Anti-tuberculosis drug resistance is a major public health problem that threatens the progress made in tuberculosis care and control worldwide. Treatment success rates of multidrug-resistant tuberculosis (MDR-TB) is a key issue that cannot be ignored. There is a paucity of evidence that assessed studies on the treatment of MDR-TB, which focus on the effectiveness of the directly observed treatment, short-course (DOTS)-Plus program. Therefore, it is crucial to assess and summarize the overall treatment outcomes for MDR-TB patients enrolled in the DOTS-Plus program in recent years. The purpose of this study was to thus assess and summarize the available evidence for MDR-TB treatment outcomes under DOTS-Plus.

**Methods:**

A systematic review and meta-analysis of published literature was conducted. Original studies were identified using the databases MEDLINE®/PubMed®, Hinari, and Google Scholar. Heterogeneity across studies was assessed using the Cochran’s Q test and I^2^ statistic. Pooled estimates of treatment outcomes were computed using the random effect model.

**Results:**

Based on the 14 observational studies included in the meta-analysis, it was determined that 5 047 patients reported treatment outcomes. Of these, the pooled prevalence, 63.5% (95% *CI*: 58.4–68.5%) successfully completed full treatment (cured or treatment completed) with a pooled cure rate of 55.6%, whereas 12.6% (95% *CI*: 9.0–16.2%) of the patients died, 14.2% (95% *CI*: 11.6–16.8%) defaulted from therapy, and 7.6% (95% *CI*: 5.6–9.7%) failed therapy. Overall 35.4% (95% *CI*: 30–40.8%) of patients had unsuccessful treatment outcomes. An unsatisfactorily high percentage 43% (95% *CI*: 32–54%) of unsuccessful treatment outcomes was observed among patients who were enrolled in standardized treatment regimens.

**Conclusion:**

This study revealed that patients with MDR-TB exhibited a very low treatment success rate compared to the World Health Organization 2015 target of at least 75 to 90%. The high default rate observed by conducting this literature review could possibly explain the spread of the MDR-TB strain in various populations. A better treatment success rate was observed among patients in individualized treatment regimens than in standardized ones. Conducting further individual-based meta-analysis is recommended to identify potential factors for defaulting treatment using large-scale and multi-center studies.

**Electronic supplementary material:**

The online version of this article (doi:10.1186/s40249-016-0214-x) contains supplementary material, which is available to authorized users.

## Multilingual abstracts

Please see Additional file 1 for translations of the abstract into the six official working languages of the United Nations.

## Background

Anti-tuberculosis (TB) drug resistance is a major public health problem, which threatens the progress made in the control of TB worldwide. Drug resistance occurs due to the improper use of antibiotics during chemotherapy of drug-susceptible TB patients. This incorrect use is a result of a number of factors such as administration of inappropriate treatment regimens and failure to ensure that patients complete the whole course of treatment [1].

Globally, 5% of TB cases were estimated to have developed multidrug-resistant TB (MDR-TB) (defined as resistance to at least isoniazid and rifampin) in 2013 (3.5% new and 20.5% previously treated TB cases). Likewise, drug resistance surveillance data have shown that an estimated 480 000 people developed MDR-TB worldwide in 2013, and out of this, 210 000 people died [1]. In 1999, the Green Light Committee (GLC), a partner of the World Health Organization (WHO), launched the “directly observed treatment, short-course (DOTS)-Plus for MDR-TB” programs for patients with MDR-TB. The program emphasizes the usage of appropriate second-line drugs (SLDs) in low- and middle-income settings. By the end of 2006, more than 50 DOTS-Plus pilot programs had been launched by GLC, and more than 20 000 patients with MDR-TB were under treatment [2].

The DOTS-Plus program, which stresses the combination of first- and second-line drugs to treat MDR-TB, is becoming increasingly important for MDR-TB control globally. The core components are comprehensive to ensure that all essential elements of the DOTS-Plus strategy are included. They are the following: sustained political and administrative commitment; diagnosis of MDR-TB through quality-assured culture and drug susceptibility testing; appropriate treatment strategies that utilize SLDs under proper management conditions; and uninterrupted supply of quality-assured anti-TB drugs [3].

Treatment of MDR-TB involves the use of SLDs, which are more complex, toxic, costly, and less effective, are associated with a greater incidence of adverse reactions [4, 5], and require longer treatment duration than first-line drugs.

The WHO aimed to achieve the target of at least 75 to 90% treatment success rate for TB patients by 2015 [6], but current studies on MDR-TB treatment reveal a huge gap in reaching this target. According to a WHO 2014 report, only 48% of patients with MDR-TB in 2011 were successfully treated, 16% died, 24% did not have their treatment outcomes documented or interrupted treatment, and 12% were not cured despite receiving treatment [1]. Hence, unsuccessful treatment of MDR-TB is a key problem that cannot be neglected. There is a paucity of evidence that assessed studies on the treatment of MDR-TB, which focus on the effectiveness of the directly observed treatment, short-course (DOTS)-Plus program [7]. Therefore, it is very important to review and summarize the overall treatment outcomes for MDR-TB under DOTS-Plus in recent years. The aim of this study was to assess and summarize the available evidence on MDR-TB treatment outcomes under DOTS-Plus.

## Methods

### Study design and data sources

A systematic review and meta-analysis of published observational studies was conducted. Original studies providing information on the treatment outcomes of patients with MDR-TB under DOTS-Plus were identified through a computerized search using the databases MEDLINE®/PubMed®, Google Scholar, Embase®, and Health InterNetwork Access to Research Initiative (Hinari). A detailed search strategy was implemented and reference lists were cross-checked. A combination of keywords and phrases, namely “tuberculosis”, “drug resistance”, “multidrug resistance”, “DOTS-Plus”, “MDR-TB”, and “treatment outcomes” were used to search for articles in the databases. *The International Journal of Tuberculosis and Lung Disease* was selected as the key journal for hand searching. At the same time, a hand search was also done for cross-reference lists from identified original articles and reviews for other relevant articles. The literature search, review, and data abstraction was performed from February 1 to August 30, 2015.

### Study selection

Observational studies obtained from the literature search were checked by title and citation. References from the selected studies were also assessed to ensure that no relevant studies were omitted. Studies were required to meet the following inclusion criteria: 1) involving culture-confirmed MDR-TB; 2) treatment outcome definitions specified by mycobacterial culture endpoints (e.g., “cured” defined as at least five consecutive negative cultures during the last 12 months of treatment); 3) clearly defined treatment protocols including SLDs; and 4) outcomes reported according to the WHO classification of success (including cure or treatment completion), failure, default (treatment interruption), and death [8]. Reports on original studies, unpublished master’s theses, and PhD dissertations written in English were also considered.

**Fig. 1:**
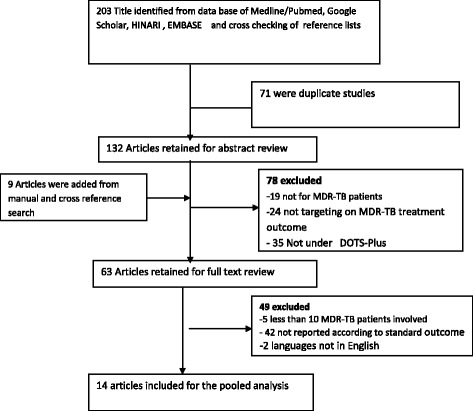
Flow chart showing the selection of studies for systematic review (identification and screening, eligible and included studies). NB: Articles may have been excluded for more than one reason

Studies were excluded from the analysis for any of the following reasons: involving patients who all had extensively drug-resistant TB; comments, editorial reviews, and articles focusing only on extrapulmonary TB; dealing with a mycobacterium other than TB; conducted among children under 15 years of age; focusing on treatment that was not under the DOTS-Plus umbrella; did not specify strategy; duplicate publications of the same study; available only in abstract form; and with a sample size of less than 10. The selection of articles for review was done in three stages: looking at the titles alone, then abstracts, and then full-text articles (see Fig. 1).

### Operationalization of outcome measures

Treatment outcomes of MDR-TB were defined based on the WHO guidelines, as follows [1]:

- Cured: defined as a patient who had completed treatment according to program protocol and who has been consistently culture-negative (with at least five results) for the final 12 months of treatment.
- Treatment completed: defined as a patient who had completed treatment according to program protocols, but does not meet the definition of “cured” because of a lack of bacteriological results.
- Died: defined as a patient who died for any reason during the course of TB treatment.
- Treatment failed: if two or more of the five cultures recorded in the final 12 months of treatment were positive, or if any one of the final three cultures was positive.
- Lost to follow-up: defined as a patient whose TB treatment was interrupted for two or more consecutive months for any reason.
- Transferred out: defined as a patient who had been transferred to another reporting and recording unit, and for whom the treatment outcome was unknown.

For the purposes of this review, those cured or who completed their treatment was categorized as successful treatment outcomes, whereas the others were categorized as unsuccessful treatment outcomes.

### Assessment of methodological quality

Studies were assessed for quality and the high-quality studies were then analyzed. The studies were considered high quality if they reported outcomes on at least 10 patients; involved culture-confirmed MDR-TB; were prospective cohort (PC), retrospective cohort (RC), or case control in design; reported an average treatment duration of ≥18 months; reported basic demographic data; and reported that less than 20% of patients were lost to follow-up. When study populations overlapped, we included the more recent and larger study population in the analysis.

### Data abstraction

Two reviewers (YM and KTK) performed data abstraction independently using a pretested standard abstraction form. The selected studies were reviewed to extract the following data: title; author(s); year of publication; study design; study site; sample size; data collection procedures; type of MDR-TB (primary, acquired); HIV status; duration of MDR-TB treatment; MDR-TB regimen (individualized treatment versus standardized treatment); response rates; and MDR-TB treatment outcomes. When there was a disagreement in data abstraction between the two investigators, it was resolved through discussion and consensus.

### Statistical analysis

Epi Data version 3.1 (EpiData Data Entry, Data Management and basic Statistical Analysis System. Odense Denmark, EpiData Association) and Stata version 11.0 (Stata Corp., College Station, Texas, USA) were used for data entry and analysis, respectively. The detail description of the original studies was presented in a table and forest plot. The pooled estimate of MDR-TB treatment was determined using the Dersimonian-Laird for random effects meta-analysis (random effects model), and was measured as proportions of treatment outcomes with 95% confidence intervals (CIs).

#### Subgroup analysis

Subgroup analyses was performed by type of treatment regimen.

#### Statistical heterogeneity and exploration of publication bias

The Begg’s rank correlation test and Egger weighted regression test were used to statistically assess publication bias, and *p* < 0.05 was considered as indicative of statistically significant publication bias.

Statistical heterogeneity between studies was evaluated using the Cochran’s Q test, which shows the amount between study heterogeneity and I^2^ statistic. The I^2^ statistic is a measure of the percentage of variability (inconsistency) between studies that happened due to by chance as conflicted to the actual difference between study populations. Therefore, the presence of statistical heterogeneity was tested using Cochran’s Q test (*P* < 0.10 indicative of statistically significant heterogeneity) and I^2^ test (values of 25, 50 and 75% were considered to represent low, medium, and high heterogeneity, respectively) [9–11].

## Results

We identified a total of 203 original articles, from the initial computer-based search, in the databases PubMed®, Hinari, and Google Scholar. Nine additional articles were identified through hand searching. Of these, 149 studies were excluded: 71 were duplicated studies, 19 did not examine patients with MDR-TB, 24 were not targeting MDR-TB treatment outcomes, and 35 did not describe treatment under DOTS-Plus. Therefore, 63 full-text studies were eligible for in-depth analysis. Here again, after a careful evaluation, two were excluded because of language restrictions (not English), 42 were excluded because they failed to report on standard outcomes, and five were excluded as they involved sample sizes of less than 10 patients with MDR-TB. Finally, the 14 remaining articles were used for the meta-analysis, including a total study population of 5047 patients from eight high-burden MDR-TB countries, namely Peru, Latvia, Estonia, Russia, the Philippines, India, South Africa, and Uzbekistan (including Karakalpakstan). Figure 1 shows how the studies were selected.

### Characteristics of studies included in the review

All of the studies selected for systemic review and meta-analysis were observational studies, 10 of which were RC studies, while four of which were PC studies. All of the studies were published in English, with study populations varying from 52 to 1 768, and undertaken between 1997 and 2013. General characteristics and description of the studies selected for the meta-analysis are outlined in Table 1.

**Table 1:** Summary of the 14 observational studies assessing the treatment outcomes for patients with MDR-TB under DOTS-Plus that were included in the meta-analysis (*n* = 14)

Study authors	Study location	Study period	Study design	Sample size	HIV (%)	Treatment duration	Treatment regimen
Kurbatova et al. [16]	Five countries^a^	2000–2003	RC	1768	1.6		Individualized
Singla et al. [17]	India	2002–2006	RC	126	–	24–27 months	Standardized
Keshavjee et al. [18]	Russia	2000–2004	RC	579	0.9	≥24 months	Individualized
Riekstina et al. [19]	Latvia	2002	RC	75	–	24 months	Individualized
Cox et al. [20]	Karakalpakstan, Uzbekistan	2003–2005	PC	87	–	≥24 months	Individualized
Shin et al. [21]	Russia	2000–2002	RC	244	0	≥12 months	Individualized
Holtz et al. [22]	Latvia	2000	RC	167	–	12–18 months after conversion	Individualized
Leimane et al. [23]	Latvia	2000	RC	204	0.5	12–18 months after conversion	Individualized
Tupasi et al. [24]	Philippines	1999–2002	PC	149	–	≥24 Months	Individualized
Arora et al. [25]	India	2002–2005	RC	52	–	24 months	Standardized
Mitnick et al. [26]	Peru	1999–2002	RC	651	1.5	15 months after conversion	Standardized
Jain et al. [27]	India	2009	PC	130	–	24 months	Standardized
Farley et al. [28]	South Africa	2000–2004	PC	757	38	12–18 months after conversion	Standardized
Van Deun et al. [14]	Bangladesh	1997–1999	RC	58	–	24 months	Standardized

^a^The five countries were Peru, Latvia, Estonia, Russia, and the Philippines

### Treatment outcomes

The lowest cure rate (21%) was reported in a study conducted in South Africa and the highest cure rate (77%) was reported in a study conducted in Russia.

**Fig. 2:**
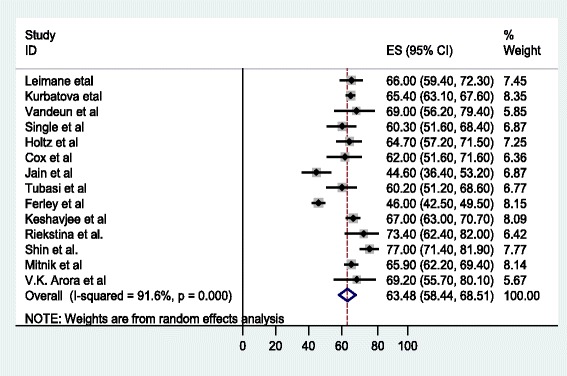
Forest plot of the 14 observational studies that quantitatively assessed successful MDR-TB treatment outcomes under DOTS-Plus

**Fig. 3:**
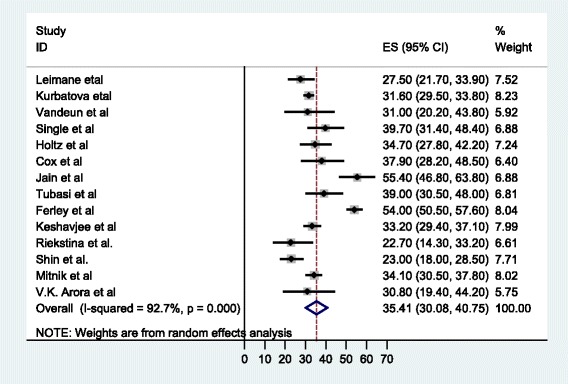
Forest plot of the 14 observational studies that quantitatively assessed unsuccessful MDR-TB treatment outcomes under DOTS-Plus

The meta-analysis of the 14 studies, in which SLDs under the DOTS-Plus program in individualized or standardized protocols were administered, determined that 5 047 patients reported treatment outcomes, with 63.5% (58.4%, 68.5) meeting the definition of successful treatment (cured or treatment completed). The pooled cure rate was 55.6%, whereas 12.6% (9.0, 16.2) of the patients died, 14.2% (11.6, 16.8) defaulted from therapy, and 7.6% (5.6, 9.7) failed therapy (see Fig. 2). The overall percentage of unsuccessful treatment outcomes was 35.4% (30, 40.8) (see Fig. 3).

**Fig. 4:**
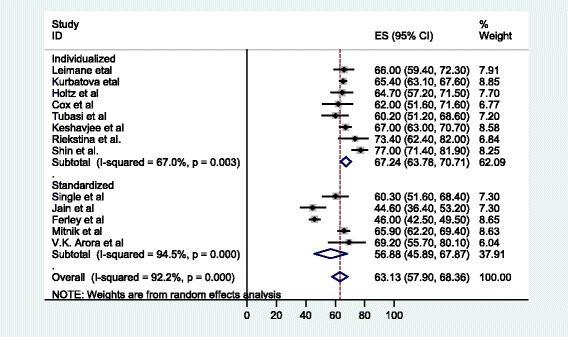
Forest plot of the 13 observational studies that quantitatively assessed successful MDR-TB treatment outcomes under DOTS-Plus by treatment regimen

**Fig. 5:**
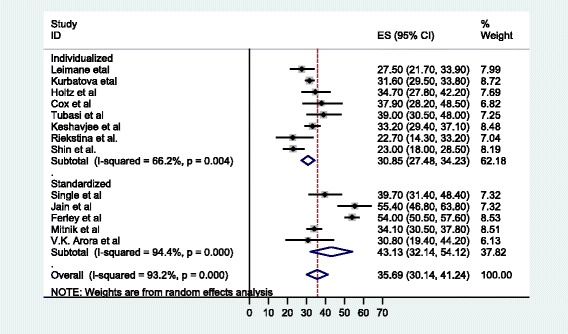
Forest plot of the 13 observational studies that quantitatively assessed unsuccessful MDR-TB treatment outcomes under DOTS-Plus by treatment regimen

According to a subgroup analysis, in the eight studies that reported on individualized treatment regimens for patients with MDR-TB, the pooled proportion of patients achieving treatment success was 67.2% (63.7, 70.7) (see Fig. 4), with a cure rate of 62.7% (57.2, 68.2). The pooled proportion of patients who had unsuccessful treatment outcomes was 30.85% (27.5, 34.2) (see Fig. 5), with treatment failure, default, and death rates of 8.4, 13 and 8.4%, respectively. On the other hand, in the five studies in which MDR-TB patients were under standardized treatment regimens, the pooled proportion of patients successfully treated was 56.9% (45.9, 67.9) (see Fig. 4), with a cure rate of 50.9% (26.8, 75). The pooled proportion of patients who had unsuccessful treatment outcomes was 43% (32, 54) (see Fig. 5), with failure, default (treatment interruption), and death rates of 6.9, 16.6 and 20.2%, respectively.

## Discussion

To determine the pooled treatment success rate of patients with MDR-TB enrolled in DOTS-Plus programs and thus receiving SLDs, we analyzed data from 14 studies in eight countries, which reported on treatment outcomes for a total of 5 047 patients. The results of this meta-analysis revealed that high proportions of patients had poor treatment outcomes. The subgroup analysis further showed that treatment success rate was higher in studies that reported on individualized treatment regimens.

Standardized treatment ensures that all patients with MDR-TB receive the same treatment regimens involving a common drug susceptibility test (DST) of the prevalent MDR-TB strains. Individualized treatment, on the other hand, highlights the provision of treatment based on a previous history of anti-TB treatment, while also taking into account DST results from the most recent isolate obtained from the patient [12, 13].

Empirically, an individualized treatment strategy for MDR-TB requires ready access to reliable laboratory facilities and to healthcare providers who are trained to prescribe the regimens and interpret the results [14]. It is likely that individualized treatment may be very difficult to administer in resource-limited settings, as compared to standardized treatment. However, individualized treatment improves selection of effective drugs that can result in high treatment success for patients and prevents the acquirement of a further resistance strain.

Our study, unlike previous reviews [7, 15], solely focused on MDR-TB treatment outcomes under the recently implemented DOTS-Plus program. Previous systematic reviews and meta-analyses of studies on MDR-TB included treatment outcomes under all programs (including DOTS, and DOTS-Plus). Our meta-analysis showed that the treatment success rate was 63.48%. This finding is consistent with two previous reviews. According to Orenstein et al. [7] and Johnston et al. [15] meta-analysis studies, the overall pooled prevalence of MDR-TB treatment success estimates were 62% (95% *CI*, 58–67%) and 62% (95% *CI*, 57–67%), respectively.

The WHO aimed to achieve at least 75 to 90% treatment success rate for TB patients by 2015 [6], and to administer better treatment for MDR-TB patients. To do this, in 1992, the WHO launched the “DOTS-Plus for MDR-TB” programs aimed at MDR-TB patients, stressing the appropriate usage of SLDs in low- and middle-income settings [2]. But the current pooled analysis showed that there are huge gaps in achieving this target and that the DOTS-Plus strategy does not result in significant changes in MDR-TB treatment success rates, this implicates that more stringent activities and mobilization of resources for the treatment and control of MDR-TB is required than ever made.

The current review and meta-analysis of 14 observational studies revealed high rates of default (14.2%) and death (12.6%). Similar findings were reported in previous systematic review studies: Johnston et al. [15] reported a 13% (9–17%) default rate and a 11% (9–13%) death rate, and Orenstein et al. [7] found that the default and death rates were 12% (8–16) and 11% (7–15), respectively. Even though the treatment success rate was encouraging, the current review revealed that the DOTS-Plus treatment strategy cannot achieve the WHO treatment success target [6].

### Limitations of the study

Our review had several limitations. We relied exclusively on observational data for treatment outcomes. This may underestimate pooled treatment outcomes. Secondly, this study only searched for studies published in English, which may have resulted in information being missed about the DOTS-Plus program for MDR-TB reported in other languages. Presence of high heterogeneity across studies is another limitation of this review.

## Conclusion

This meta-analysis revealed that a low MDR-TB treatment success rate among patients, which is far from the WHO target to be achieved by 2015. A significant proportion of patients defaulted from treatment (14%), or died (12.6%), which is a serious public health concern that needs to be addressed urgently. The high default rate could possibly explain the spread of the MDR-TB strain in various populations. Better treatment success rates were observed among patients in individualized treatment regimens rather than in standardized ones. Further individual-based meta-analysis is recommended to identify potential factors for defaulting treatment using large-scale and multi-center studies.

## Additional file

Additional file 1: Multilingual abstracts in the six official working languages of the United Nations. (PDF 662 kb)

## Abbreviations

CI: Confidence interval
DOTS-Plus: Directly observed treatment, short-course-Plus
DST: Drug susceptibility test
GLC: Green Light Committee
Hinari: Health InterNetwork Access to Research Initiative
MDR-TB: Multidrug-resistant tuberculosis
PC: Prospective cohort
RC: Retrospective cohort
SLD: Second-line drug
TB: Tuberculosis
WHO: World Health Organization
