# Supplementary material for: Treatment outcomes for multidrug-resistant tuberculosis under DOTS-Plus: a systematic review and meta-analysis of published studies
Source: Infect Dis Poverty. 2017 Jan 17;6:7. doi: 10.1186/s40249-016-0214-x (PMC5240443; doi:10.1186/s40249-016-0214-x)
Supplement: Additional file 1: — Multilingual abstracts in the six official working languages of the United Nations. (PDF 662 kb) [file 40249_2016_214_MOESM1_ESM.pdf]

Translation of the abstract into the six official working languages of the United Nations

نتائج علاج السل المقاوم للأدوية المتعددة في إطار المعالجة قصيرة الأمد (المعدلة) تحت الاشراف المباشر: مراجعة منهجية وتحليل تلوي للدراسات المنشورة

Kelemu Tilahun Kibret, Yonatan Moges, Peter Memiah, Sibhatu Biadgilign

ملخص

خلفية: تعد مشكلة مرض السل المقاوم للأدوية مشكلة صحية عامة رئيسية تهدد التقدم المحرز في مجال علاج مرض السل ومكافحته على مستوى العالم. ولذلك فإن معدل نجاح علاج السل المقاوم للأدوية المتعددة يعد مشكلة رئيسية لا يمكن تجاهلها. يوجد نقص في الأدلة التي تقييم الدراسات على علاج السل المقاوم للأدوية المتعددة والتي تركز على فعالية المعالجة قصير الأمد (المعدلة) تحت الاشراف المباشر. وبالتالي من الضروري تقييم وتلخيص نتائج العلاج بأكلمه للمرضى الذين يعانون من مرض السل المقاوم للأدوية المتعددة والمسجلين في السنوات الأخيرة في برنامج المعالجة قصيرة الأمد (المعدلة) تحت الاشراف المباشر. وبالتالي كان الهدف من هذه الدراسة تقييم وتلخيص الأدلة المتوفرة لنتائج علاج السل المقاوم للأدوية المتعددة في إطار برنامج المعالجة قصيرة الأمد (المعدلة) تحت الاشراف المباشر.

الأساليب: أجريت مراجعة منهجية وتحليل تلوي للدراسات المنشورة. حُددت الدراسات الأصلية باستخدام قواعد البيانات المنشورة في محركات البحث ميدلاين/ بميد وبرنامج Hinari والباحث العلمي من جوجل. وجرى تقييم عدم التجانس عبر الدراسات باستخدام اختبار كوكران واحصاء I^2 · تم حساب التقديرات المجمعة من نتائج العلاج باستخدام نموذج تأثير عشوائي.

النتائج: واستنادًا إلى الأربعة عشر دراسة رصدية المدرجة في التحليل التلوي، تبين أن 5,047 مريض قد أبلغ عن نتائج العلاج. ومن بين هؤلاء، أكمل أعداد محددة من المرضى المجمعين في وقت محدد، بلغت نسبتهم63.5% (95% التحقيق السريري: 58.4-68.5%) العلاج كاملاً بنجاح (تم علاجهم أو شفائهم بالكامل) حيث وصلت نسبة الشفاء المجمعة إلى 55.6% في حين بلغ عدد المرضى الذين لقوا حتفهم 12.6% (95% التحقيق السريري: 9.0-16.2%)، أما المرضى المتخلفين عن العلاج فقد بلغوا 14.2% (95% التحقيق السريري: 11.6-16.8%) أما المرضى الذين فشلوا في العلاج فقد بلغت نسبتهم 7.6% (95% التحقيق السريري: 5.6-9.7%). وبوجه عام بلغ عدد المرضى الذين كانت نتائج علاجهم غير ناجحة 35.4% (95% التحقيق السريري: 30-40.8%). وقد لوحظ نسبة عالية غير مرضية بلغت 43% (95% التحقيق السريري: 32-54)%) من نتائج العلاج غير الناجحة بين المرضى الذين كانوا مسجلين في نظم العلاج الموحدة.

الخلاصة: كشفت هذه الدراسة بأن المرضى الذين يعانون من السل المقاوم للأمراض المتعددة قد أظهروا نسبة نجاح منخفضة جداً للعلاج مقارنة مع هدف منظمة الصحة العالمية عام 2015 وهو ألا تقل نسبة نجاح العلاج من 75% إلى 90%. إن نسبة التخلف العالية التي تمت ملاحظتها من خلال استعراض المنشورات يمكن أن تشرح سبب انتشار سلسلة مرض السل المقاوم للأمراض المتعددة بين الشعوب المختلفة. وقد لوحظ ارتفاع نسبة نجاح العلاج بين المرضى في نظم العلاج الفردية مقارنة بنظم العلاج الموحدة. ويوصي بإجراء المزيد من التحاليل التلوية على أساس فردي لتحديد العوامل المحتملة التي تسبب التخلف عن العلاج وذلك باستخدام الدراسات واسعة النطاق والدراسات التي تجرى بين عدة مراكز.

Translated from English version into Arabic by Yosra Montasser, through

TRANSLATORS WITHOUT BORDERS

DOTS-Plus 项目对多重耐药性结核病的疗效：对已有研究的系统综述和荟萃分析

Kelemu Tilahun Kibret, Yonatan Moges, Peter Memiah, Sibhatu Biadgilign

摘要

引言：抗结核药物的耐药性是一个威胁全球结核病治疗和控制进展的重大公共卫生问题。多重耐药性结核病(MDR-TB)的治疗成功率是一个不容忽视的关键议题。关于多重耐药性结核病治疗的评估研究，尤其是对直接观察治疗和直接面视下短程化学疗法（DOTS）-Plus 的有效性的研究极少。因此，评估和总结近年来参加 DOTS-Plus 项目的多重耐药性结核患者的整体治疗结果具有重要意义。本研究的目的是基于现有证据评估和总结基于 DOTS-Plus 框架下多重耐药性结核病的疗效。

方法：本研究通过对已发表文献的系统回顾和荟萃分析，使用 MEDLINE®/PubMed®、Hinari 和 Google Scholar 数据库确定原始研究。采用 Cochran's Q test 和 I^2 statistic 方法评估研究异质性。采用随机效应模型估算总疗效。

结果：根据对 14 个观察性研究的荟萃分析发现，共计有 5 047 例病例报告了治疗结果。其中，总的患病率为 63.5% (95% *CI*: 58.4- 68.5%)，成功接受完整治疗（治愈和完整治疗）的总治愈率为 55.6%，然而有 12.6% (95% CI: 9.0-16.2%)的病例死亡，14.2% (95% CI: 11.6-16.8%)病例未完成治疗，7.6%

(95% *CI*: 5.6-9.7%)病例治疗失败。超过 35.4% (95% *CI*: 30-40.8%)的病例治疗结果不成功。在参加标准治疗方案的患者中观察到的不成功治疗结果高达 43% (95% *CI*: 32-54%)。
结论：本研究揭示多重耐药性结核病例的成功治愈率与世界卫生组织 2015 年达到至少 75%-90%的要求相比存在较大差距。文献综述中出现的较高的违约率或许可用于解释在人群中不断扩散的多重耐药性株。与标准化治疗相比，个性化治疗的成功治疗率更好。建议进一步开展基于个性化治疗的荟萃分析来确认在大规模多中心研究处理违约的潜在要素。

Translated from English version into Chinese by Xin-Yu Feng, edited by Pin Yang

Résultats thérapeutiques du traitement de la tuberculose multi-résistante dans le cadre du programme DOTS-Plus: une revue et une méta-analyse systématiques d’études publiées

Kelemu Tilahun Kibret, Yonatan Moges, Peter Memiah, Sibhatu Biadgilign

RÉSUMÉ

Contexte: la résistance aux médicaments anti-tuberculiques constitue un important problème de santé publique qui porte préjudice aux avancées obtenues dans la prise en charge et la lutte contre la tuberculose dans le monde entier. Le taux de réussite du traitement de la tuberculose multi-résistante est une question fondamentale qui ne peut plus être ignorée. Il faudrait bien plus de données factuelles relatives à l’évaluation d’études sur le traitement de la tuberculose multi-résistante et concentrées sur l’efficacité du programme de traitement sous observation directe de courte durée (DOTS)-Plus. Il est donc essentiel d’évaluer et de résumer les résultats thérapeutiques globaux des patients atteints d’une tuberculose multi-résistante inclus au programme DOTS-Plus au cours des dernières années. L’objectif de la présente étude consistait ainsi à évaluer et à résumer les données factuelles disponibles pour les résultats de traitement de la tuberculose multi-résistante dans le cadre du programme DOTS-Plus.
Méthodes: une revue et une méta-analyse systématiques de la littérature publiée a été menée. Des études originales ont été identifiées en utilisant les bases de données MEDLINE®/PubMed®, Hinari et Google Scholar. L’hétérogénéité parmi les études a été évaluée à l’aide du test Q de Cochran et à la statistique I^2. Les estimations groupées des résultats de traitement ont été calculées en utilisant le modèle à effet aléatoire.
Résultats: nous avons déterminé que 5047 patients avaient fait état de résultats thérapeutiques sur la base des 14 études d’observation comprises dans la méta-analyse. Sur ces derniers, la prévalence globale, 63,5% (*IC* à 95%: 58,4-68,5%) ont achevé avec succès un traitement complet (guérison ou traitement achevé) à un taux de guérison global de 55,6%, tandis que 12,6 % (*IC* à 95%: 9,0-16,2%) des patients sont décédés, 14,2%(*IC* à 95%: 11,6-16,8%) ont interrompu le traitement et 7,6% (*IC* à 95%:5,6-9,7%) n’ont pas obtenu de bons résultats. Au total, 35,4% (*IC* à 95%: 30-40,8%) des patients présentaient des résultats thérapeutiques infructueux. Une proportion élevée et insatisfaisante de 43% (*IC* à 95%: 32-54%) d’échecs de traitement a été observée parmi les patients suivant des schémas thérapeutiques standardisés.
Conclusion: cette étude a révélé que les patients atteints d’une tuberculose multi-résistance présentaient un taux de réussite du traitement très faible par rapport à l’objectif 2015 de l’Organisation Mondiale de la Santé d’au moins 75% à 90%. L’important taux de défaillance constaté lors de la réalisation de cette revue de la littérature pourrait éventuellement expliquer la propagation de la souche multi-résistance de la tuberculose au sein de populations variées. Un meilleur taux de réussite du traitement a été observé parmi des patients suivant des schémas thérapeutiques personnalisés par rapport à ceux suivant des schémas standardisés. La réalisation d’une méta-analyse approfondie reposant sur les individus est recommandée pour identifier les facteurs potentiels de défaillance de traitements sur la base d’études de grande envergure et multicentriques.

Translated from English version into French by eric ragu, through

Результат лечения многомедикамент-сопротивляющегося туберкулеза под метод DOTS-Plus: Это систематический обзор и мета-анализ для опубликованного исследования.

Kelemu Tilahun Kibret, Yonatan Moges, Peter Memiah, Sibhatu Biadgilign

Реферат

Фон: Сопротивляемость медикамента анти-туберкулеза является главной публичной проблемой, которая грозит прогресс в сфере заботы и контроля туберкулеза в мире. Отношение успеха лечения многомедикамент-сопротивляющегося туберкулеза (MDR-TB) является ключевым исходом, чему нужно уделять внимание. Невозмоно оценить исследование по лечению MDR-TB без достаточных данных. Учение сосредоточится в эффективности лечения под прямое наблюдение, это краткосрочная программа DOTS-Plus. Следовательно, важно оценить и суммировать всеобщий результат лечения для пациентов с MDR-TB, которые записывали в программу DOTS-Plus в последние годы. Таким образом, цель этой работы заключится в том, чтобы оценить и суммировать доступные данные для результата лечения MDR-TB под метод DOTS-Plus.

Методы: Печатный материал связывается с принципами “систематический обзор и мета-анализ”. Оригинальное исследование пользовало базу данных “ MEDLINE®/PubMed®, Hinari и Google Scholar. При исследовании, гетерогенность была оцененна пользовать Cochran’s Q тест и статистический I^2.

Результаты: Мета-анализ включает в себя 14 наблюдательных исследований, который показывает отчёт результата лечения 5047 пациентов. Среди них, суммарная общепринятость, 63.5% (95% *CI*: 58.4- 68.5%) успешно совершили полное лечение (были излеченны или совершили лечение) с 55.6% суммарной излечимостей, но 12.6% (95% *CI*: 9.0-16.2%) из пациентов умерли, 14.2% (95% *CI*: 11.6-16.8%) не соблюдали метод лечения и 7.6% (95% *CI*: 5.6-9.7%) потерпели неудачу метода лечения. 35.4% (95% *CI*: 30-40.8%) из пациентов получили неудачный результат лечения. Среди пациентов, которые вступили в стандартизованное лечение режима медицины, 43% (95% *CI*: 32-54%) из них получили неудачные результаты лечения.

Заключение: В этом исследовании оказалась низкая излечимость для пациентов с MDR-TB, по сравнению с целевой показателей (не менее 75%-90%) ВОЗ. Высокая показатель невыполнения была наблюдённа этом литературным обзором возможно объяснить распространение MDR-TB между различными населениями. Более высокая излечимость появилась когда пациенты приняли личные лечения режима медицины вместо стандартизованных. Рекомендую дальше вести мета-анализ личного-базиса, чтобы определить потенциальные факторы для лечения невыполнения под масштабных и многомеди-центральных исследований.

Translated from English version into Russian by Hao-qi Zhang

Resultados del tratamiento de la tuberculosis multi-resistente a los fármacos en el programa DOTS-Plus: revisión sistemática y meta-análisis de los estudios publicados

Kelemu Tilahun Kibret, Yonatan Moges, Peter Memiah, Sibhatu Biadgilign

RESUMEN

Antecedentes: La resistencia a los fármacos anti-tuberculosos constituye un problema central de la salud pública que compromete el avance logrado en el control y atención de la tuberculosis en todo el mundo. La tasa de tratamiento exitoso de la fármaco-resistencia múltiple para la tuberculosis (MDR-TB) es crucial y no puede ser ignorada. De la evaluación de los estudios surgen evidencias insuficientes sobre el tratamiento para la MDR-TB, que se centra en la eficacia del tratamiento observado directamente, el programa de corta duración DOTS-plus. Por lo tanto, es crucial evaluar y sumariar los resultados del tratamiento completo para pacientes MDR-TB enrolados en el programa DOTS-Plus en los últimos años. El propósito de este

estudio fue, entonces, evaluar y sumariar la evidencia disponible sobre los resultados del tratamiento MDR-TB en el programa DOTS-Plus.
Métodos: Se realizó la revisión sistemática y el meta-análisis de la literatura publicada. Los estudios originales fueron identificados utilizando las bases de datos MEDLINE®/PubMed®, Hinari y Google Scholar. Para cuantificar la heterogeneidad entre los estudios se ha utilizado el test Q de Cochran y el parámetro estadístico I^2. Las estimaciones de los resultados del tratamiento se calcularon utilizando el modelo de efectos aleatorios.
Resultados: Sobre la base de 14 estudios observacionales incluidos en el meta-análisis se determinó que 5.047 pacientes reportaron resultados del tratamiento. De ellos, el grupo dominante, el 63,5% (IC del 95%: 58.4- 68.5%) completó con éxito un tratamiento completo (se curaron o completaron el tratamiento) con una tasa de curación del conjunto de 55,6%, mientras que el 12,6% (IC del 95%: 9,0 a 16,2%) de los pacientes fallecieron; el 14,2% (IC del 95%: 11,6-16,8%) incumplió con la terapia, y el 7,6% (IC del 95% : 5.6 a 9.7%) fracasó en la terapia. Un elevado porcentaje, y por lo tanto insatisfactorio, del 43% (IC del 95%: 32-54%) de los resultados de los tratamientos fallidos se observó entre los pacientes que fueron incluidos en los regímenes de tratamiento estandarizados.
Conclusiones: El estudio reveló que los pacientes con MDR-TB mostraron una tasa de tratamiento exitoso muy baja comparada con la meta de la Organización Mundial de la Salud de 2015 fijada en, al menos, el 75% al 90%. La alta tasa de incumplimiento observada mediante la realización de esta revisión de la literatura podría explicar, posiblemente, la propagación de la cepa MDR-TB en diversas poblaciones. Se observó una tasa de tratamiento exitoso entre los pacientes en regímenes de tratamiento individualizado en comparación con los enrolados en regímenes de tratamiento estandarizados. Se recomienda realizar nuevos meta-análisis basados en los individuos a fin de identificar los factores potenciales que llevan al incumplimiento del tratamiento utilizando estudios a gran escala y en múltiples centros.

Translated from English version into Spanish by Guadalupe Barua, through

TRANSLATORS
WITHOUT BORDERS
